# Transactions American Association of Obstetricians and Gynecologists

**Published:** 1894-11

**Authors:** 


### Society Proceedings.

### TRANSACTIONS AMERICAN ASSOCIATION OF OBSTETRICIANS AND GYNECOLOGISTS.

*Seventh Annual Meeting, Toronto, September 19, 20, 21, 1894.*

(Abstract.)

[*From American Journal of Obstetrics, November, 1894*]

### APPENDICITIS.

### FIRST DAY—AFTERNOON SESSION.

DR. GEORGE S. PECK, of Youngstown, O., read a paper entitled APPENDICITIS: REPORT OF SEVEN CASES SURGICALLY TREATED, FOUR OF WHICH WERE OPERATED IN THIRTY-SEVEN CONSECUTIVE HOURS.

CASE I.—Operation during interval between attacks. Obstruction on the 6th of July. Did second operation. Operation July 27, 1894, appendix buried in mass of strong adhesions between ileum and cecum containing a large fecal concretion. Appendix removed in segments. Ileum returned to the abdominal cavity. During the first six days highest temperature, 100° ; pulse, 100. On the fifth day four attacks of vomiting, the fourth containing fecal matter. At 2 A. M., August 4th, reopened the incision and found about three feet from the ileo-cecal valve complete obstruction by a band of dense adhesions. Obstruction liberated ; ileum brought out in the incision and abdominal cavity packed with gauze. Put patient to bed at 5 A. M. ; pulse, 124 ; external heat applied and hypodermics of strychnia and digitaline every two hours. During the entire day of August 1st pulse ranged from 140 to 160 ; vomiting of fecal matter continued at frequent intervals ; tympanites increasing. Seven P. M., symptoms of obstruction continued, becoming worse every hour. As a temporary expedient, made a small opening in the ileum ; fecal matter and flatus came away in large amount, vomiting ceased, and tympanites entirely disappeared. From

August 2d to the 14th, pulse ranged from 104 to 118; passed a large amount of fecal matter through artificial anus. From August 1st to the 11th passed flatus per rectum but twice, very small quantity each time. August 11th passed well-formed fecal matter per rectum first time since operation. On the 19th day the temperature commenced to rise, and there was considerable fluctuation up to the 51st day, when it became normal and so continued. On the 28th day an unsuccessful attempt to close the fecal fistula was made; a second unsuccessful attempt was made on the 35th day. From the 13th day to the present time she has had from one to three daily passages per rectum; sat up for the first time on the 33d day, walked about the room on the 38th, and out doors every day since September 3d. Discharged from hospital on the 55th day after first operation; fistula closed.

Case II.—Operation during 4th day of first attack. Large appendix removed, containing two drams of pus and a fecal concretion. Adhesions broken up, and incision packed with iodoform gauze. Uninterrupted recovery. Discharged from hospital on the 28th day after operation.

Case III.—Operation 3d day of the third attack. Peritoneal cavity opened and adhesions broken up; large appendix removed. Uninterrupted recovery; discharged on the 27th day after operation.

Case IV.—Operation during 10th day. Death from septic peritonitis in sixty-five hours. Large abscess cavity evacuated; appendix gangrenous and detached; washed out by irrigation. Autopsy four hours after death, revealed general septic peritonitis.

Case V.—Perforating appendicitis. Operation during 3d day of attack. Death from septic peritonitis in twenty-seven hours thereafter. Appendix perforated twenty-eight hours before operation. Second operation twenty-four hours later. Median incision, obstruction removed; died from shock half an hour after operation. Autopsy immediately after death revealed extensive peritonitis with pus sac deep in the pelvis and recto-vesical pouch, containing a pint of pus.

Case VI.—Operation 4th day of attack. Death in four hours after operation from shock and septic peritonitis; appendix gangrenous throughout its entire length. Autopsy six hours after death revealed extensive general septic peritonitis.

Case VII.—Operation. Median incision; small intestines adherent to parietal peritoneum and matted together. Adhesions broken up; ileum matted together and when separated measured three feet. In liberating adhesions ileum torn in two about fifteen inches from ileocecal valve. End to end anastomosis by Murphy button. All adhesions liberated; appendix not found; thorough irrigation. Patient put to bed with a pulse of 150. Passed flatus in twelve hours; bowels moved freely on the afternoon of the second day. Beginning with the 4th day temperature normal, and pulse 96.

### APPENDICITIS WITH OBSERVATIONS BASED ON A CLINICAL STUDY OF EIGHTY-FOUR CASES.

This was the title of a paper read by Dr. W. G. MACDONALD, of Albany, N. Y. The author said that every department of abdominal surgery has its evolutionary period. The surgery of the right iliac fossa following the rule, is now drawing to the close of that period. Out of the great amount of literature, controversial and otherwise, three important landmarks are established :

1. That for all practical purposes all inflammatory processes in the right iliac fossa arise from the vermiform appendix.

2. That practically the vermiform appendix is always intraperitoneal, and that any operation undertaken for appendicitis that does not involve the entering of the peritoneum is false in its surgical conception.

3. That idiopathic peritonitis does not occur. That many cases diagnosticated as such, are really cases of perforating appendicitis.

From a careful consideration of pathological conditions and clinical histories, appendicitis may be classified in the following varieties:

1. Acute perforating, fulminating appendicitis with general peritonitis.

2. Acute suppurating appendicitis with local plastic peritonitis and abscess.

3. Subacute appendicitis, variously termed catarrhal, chronic, relapsing or obliterating appendicitis or appendicular colic (Talman).

It is not claimed that pathologically these conditions are absolutely distinct. Quite the contrary is true, but that the type is determined by the amount of bacteriological invasion and the degree of immunity possessed by the patient. That perforation occurs very much earlier than is commonly believed, can be demonstrated by many clinical histories and early operations. It may mark the onset of the disease. That the prognosis in acute perforating appendicitis, with or without operation, is always grave; that operations undertaken while perforation is impending, but has not occurred, are followed by fatal results by extension of inflammation in many cases ; that acute suppurative appendicitis with local peritonitis presents the most favorable field for operation during the attack, are well-demonstrated observations. The removal of the appendix is to be undertaken with great circumspection when it lies in the wall of an abscess cavity.

The third group of cases do not require operation during the first attack, but if repeated attacks occur, operation during quiescence is demanded. Operative results in these cases are most favorable.

Errors in diagnosis are yet common, and arise from two causes—namely, carelessness in treating abdominal diseases without physical examination; and mistaken pathological notions.

The profession has awakened, and neglected cases are much less common than formerly. That, in the future, cases will be seen and operated upon earlier, and the results will be correspondingly improved is believed.

Dr. Joseph Hoffman, of Philadelphia, followed with a paper entitled

PUS IN THE PELVIS WITH A SPECIAL REFERENCE TO APPENDICITIS AND ITS TREATMENT.

He said pus in the abdominal and pelvic cavities has been treated and considered with far more leniency than pus anywhere else in the human economy. It is the tramp manifestation of all disease. Like death, it has all times and seasons and places for its own, and too often like sleep is the twin sister of death.

Other diseases and disorders have manifestations peculiar to themselves, but pus may be the product or resultant of every disease, broadly speaking. It was long before this was a recognized fact in reference to the abdominal cavity. Women died with pints of pus bathing their viscera without its presence ever having been suspected, and idiopathic peritonitis held sovereign sway in death certificates and pathology. Once it was discovered that pus might get into the abdominal and pelvic cavities, then the wise men in physic began to discover reasons why it ought to get out of itself, just as it got in, and the let-alone doctrine held sway, with opium and poultices for its viceroys.

If there is pus in the liver we are often left to surmise it, though the exploring needle is a safe means of diagnosis. But the general manifestations of pus are not to be considered except incidentally in this contribution, and we will consider briefly the various organs in which pus makes its appearance. In the order of its probable frequency may be mentioned the kidneys, appendix vermiformis, tubes and ovaries, liver, pancreas and spleen.

In the kidneys its symptomatology is different according to the cause. If from a stone in the pelvis of the organ, the usual manifestations of pain and renal colic are present previous to suppuration, while if the origin is from the urinary tract by the transmission of purulent matter up through the bladder along the ureters, at last reaching the kidney, there is a previous history either of general disease, cystitis from some cause, enlarged prostate with retention, or chronic cystitis from stone and suppuration. Either one of these causes has a perfectly distinct history, and the treatment, with the exception of the removal of the stone from the kidney, is identical.

If an abscess goes on to rupture into the peritoneal cavity, operation can only give the slightest chance of success when done at once. So also into the other cavities, the pleura and pericardium. It must also be remembered that abscess of the liver may simulate the same disease of the kidney, and that the disease of the latter organ has been mistaken for that of the former. The presence of abscess of the spleen, as well as of the pancreas, is so rare that diagnostic features are wanting except in a general way. The nature of the organs is such that when abscess is present, it must soon make its way into the peritoneal cavity, and then from the symptoms of peritonitis as they exist under other circumstances, operation will be indicated and begun most probably as exploratory, and end with the removal of the offending organ. Pus as a foreign element is most common in the pelvic organs. Appendicitis is a most frequent cause of the trouble, and alongside of tubal and ovarian diseases it is the most prolific of causes. The diagnosis of pus as a concomitant of appendicitis is not always an easy matter.

The history of cases is that where one case escapes without suppuration, where no effort is made to save the patient, a vast number do not. So it is in recurrent attacks of appendicitis. Those who have most studied the condition, who have watched patients said to have recovered, know that in a great majority of instances they finally go into other hands, and either die in an attack or escape by operation. Operation in these forlorn cases has its justification only in the hope that a saving chance is offered an otherwise necessarily hopeless case.

Considering that the uterus is the starting-point of the pus infection, physicians anticipate the clearing out of the tubes and ovaries when infected by the curetting of the uterus. This idea is worthy of as little sound consideration, no matter by whom advanced, as the vagaries of a madhouse brain. It has no common sense to originate it, and it has not a surgical argument to support it. If our firemen were to insist on pouring water into a cellar because a conflagration originated there, paying no heed to the spread of the ruin along the roofs from house to house; if they insisted because there was already trouble and a blaze above, they might still add combustibles to the fire in the cellar, we would label them either as incendiaries, or idiots, or madmen. They would be stopped, compelled to lay aside their uniform and get out of the service. The surgical service of a long-suffering race of women needs some such police regulation to protect them from surgery which has no better sense or justification than all this.

The rules to be laid down for the relief of pus in the abdomen are to get at the point of suppuration, the cause and remove it. If this is impossible, as it is in some cases of appendicitis, and the peritoneal cavity is shut off by adhesions, the evacuation of the pus by incision over the abscess is the best procedure to save the life of the patient, and that is all we are after. If the patient is in a condition to find and remove the appendix, so much the better, but the best thing is to work so that life can be saved. In appendicitis we have one of the most trying operations in surgery, for the simple reason that the relation of the parts is not constant, and he who operates expecting to find the appendix, by McBurney's or any other point, will be woefully disappointed. In the presence of pus in the pelvis, apart from the appendix, when the ovaries and tubes are involved, the best operation is by all odds the complete one. Remove the offending organs and these in the great majority of cases can be removed by the experienced hand. If the hand is not experienced, it had better be out of the case. In the presence of hemorrhage, gauze or sponge packing is a valuable aid in controlling the blood. In the presence of pus, gauze packing is to be deprecated unless to shut off the peritoneal cavity from infection. In these cases it is only to be used if we are satisfied that there is more risk without it than with it. Gauze will not drain pus; it will only hold it in.

Treat each case according to the demands it makes and the complications. There is no rule in doing an ideal operation and have the patient dead when it is over. Ideal work is that which gives the best result in the line for which it is done. Surgery of the abdomen is a work of self-denial, of trial, of unexpected complications and lurking disaster.

Discussion on the above papers was postponed until the evening session.

FIRST DAY—EVENING SESSION.

The discussion of the three papers on appendicitis was proceeded with, the opening remarks being made by Dr. Morris.

Dr. ROBERT T. MORRIS, New York: I am not inclined to say much this evening on the subject of appendicitis. Most of you are familiar with what I have said and written on the subject from time to time. I would say, however, that I am opposed to and dislike the classification of cases as men classify them today. Any man of a scientific turn of mind feels grieved over classifications. Most men who have been trying to classify cases of appendicitis have classified them from their ultimate symptoms. I believe appendicitis is an infective, exudative inflammation of the appendix vermiformis. I believe, furthermore, that classifications can be elaborated to the extent of a person's knowledge of the English language, so that eventually the man who is well qualified in the use of this language will have a separate name for each symptom as it appears and can place it as a case of this or that sort. There is no natural elaborate classification of this disease, but simply an artificial one that each man will present for adoption, but it will not be adopted eventually by others because it is artificial.

*Mistakes in diagnosis.* I think men who are engaged in making diagnoses of appendicitis very seldom made a mistake. I have had occasion, as most of you know, to see a great many cases of this disease and have removed a good many appendices, and though I do not profess to have more diagnostic acumen than my *confrères*, I yet have been misled in one case of tuberculosis of the appendix, and in another of carcinoma of the appendix in which a year or two after an attack of typhoid fever I removed a normal appendix from among adhesions which I believe had been caused by chronic appendicitis. These are the only cases in which I have been deceived, and I think very few men who are accustomed to see these cases will be mistaken except occasionally by a case of tuberculosis or of carcinoma of the appendix, or a case in which we have adhesions from septic disease of the tube or ovary or of other structures.

As to the analysis of foreign bodies, it is quite true we do not find cherry seeds, grape seeds and various other seeds in the lumen of the appendix. I have had a pretty large collection of specimens carefully examined and analyzed, and I find more frequently than anything else calculi consisting of calcium phosphate and a little inspissated fecal matter and fat. The proportion of fat was very great in several of the specimens examined. It is rather difficult to account for this proportion of fat, especially as some of the concretions were large, and as the lumen of the appendix was cut off from the lumen of the cecum ; it occurred to me that possibly it may be due to retrograde metamorphosis of the lymphoid cells of the appendix. To determine the amount of fat in the lymphoid coats, it was found that the proportion was 8 and a fraction per cent. Three appendices were examined with small ulcerating spots, and the proportion was 14 per cent. of fat. Three others examined in which the anterior coat was ulcerating, and 26 per cent. of fat was found, showing the very large amount of fat that was being produced by slow ulceration as a result of retrograde change in the lymphoid cells in the chronic ulcerative cases. In a large proportion of cases we found a slow ulcerative process going on, because the tissues were exposed to putrefactive bacteria, to the bacteria of suppuration, not necessarily in the tissues but in the lumen of the appendix.

With reference to the question as to whether we had better separate adhesions, it had better be determined by the operator himself who knows the results following his particular technique. Every man in surgery is a law unto himself. If I were to practise drainage as Dr. Price practises it, I could not obtain the results that he does. But, personally, I separate the adhesions in almost every case of appendicitis upon which I operate. I do this in searching for other collections of pus. I do it to separate loops of bowel held in bad position by adhesions which would strangulate the bowel and to prevent re-adhesion in a bad position. I believe that can be done, but it would be unsafe to teach this. I would not dare to teach it, but I would do it myself. I believe it is the correct thing for us to do.

Dr. Joseph Price, of Philadelphia: The remarks of Dr. Morris on appendicitis to which we have just listened are the best I have ever heard, and his closing remarks are quite sufficient. We may all go home without any further discussion of the subject. I agree with him excepting in one point—namely, that he would not dare teach what he would do himself in these cases, and to verify and emphasize that point I will simply allude to the statistics of one of the papers. For instance, in speaking of the separation of adhesions and delivering the appendix, or in alluding to the recurrence of cases, the author gives two fatal cases in twenty. That of itself should be a sufficient argument for surgeons to follow the practice advocated by Dr. Morris of completing all operations, removing the cheesy, disorganized appendix, and breaking up all adhesions. I am sorry Dr. Morris does not stand out and insist upon others doing just what he can and does do so successfully.

I will allude very briefly to the carelessness in connection with surgery. A short time ago a patient died in the Pennsylvania Hospital. Dr. Morton insisted upon a section for appendicitis, but several other physicians did not agree with him. At the autopsy, in searching for the appendix a huge abscess was found about it and the appendix was necrotic. Even at post-mortems some practitioners do not find the diseased appendix, and they are at a loss to know what killed their patients. This has been so for many years. I have had a large experience in dealing with recurrent cases of appendicitis that have been recognized as such by a number of intelligent physicians in the Adirondacks, in Europe and at watering places, who insisted upon having the appendices removed. Some of these cases have come to Philadelphia, and I have found the physicians correct in their diagnoses.

Dr. Morris has alluded to foreign bodies, and I am rather inclined to think that the variety of foreign bodies found in the appendix is much greater than is ordinarily supposed. A short time ago a Philadelphia surgeon found a fish-fin in the appendix, and another gentleman found two sugar-coated pills. We know that cases of appendicitis increase about the strawberry and grape season. A few years ago an intelligent gentleman, a man of considerable wealth and leisure, traveled around the world and had twenty-four attacks of the disease in different parts of the world. He was treated by twenty-four or more physicians and by some declared cured, and in the twenty-fifth attack Dr. Agnew removed the appendix, and the man recovered. I presume Dr. Morris has a goodly number of appendices in bottles that have been reported as cured and are still being reported as such before county, state and national medical societies. My brother has had fourteen operations for appendicitis without a death. The disease is common. I have operated twice myself in one physician's family.

Dr. A. H. Cordier, Kansas City, Mo.: It is with some little timidity that I rise to participate in this discussion after hearing the able and interesting remarks of Drs. Morris and Price. I have seen a great deal of the work of Dr. Price and I have done a little of this class of work myself, and must beg leave to differ from them in regard to breaking up adhesions in this locality. I wish to call attention to the fact that there is a great deal of difference between pus found in the appendix and that in the pelvis. If we accept the theory advanced by Dr. Price that the majority of cases of pelvic suppurative diseases are caused by gonorrhea, we must accept the fact that this is a form of poisoning brought about by the gonococcus of Neisser. It does not invade the peritoneum to the same extent, and it is not as dangerous a microörganism as the bacillus coli communis. We have a different septic germ in this locality. If we go to work and break up the adhesions in a case of appendicitis that are walled off, we are sure to have a larger mortality than if we simply make an incision, evacuate the pus, clean out the cavity, and introduce rubber drainage in addition to gauze packing. In these cases it is hard to tell when to go ahead and when to stop. In some cases the appendix will float out with the pus at the time of the operation. My results in operating for cases of appendicitis have been extremely satisfactory to me. Where I find them with walled off abscesses, I simply make an incision, evacuate the pus, and drain as in other localities.

I wish to speak now of the symptom—colic. We pay too little attention to colic occurring in the abdomen. A patient presents himself with colic occurring from year to year, and it is more significant than we are disposed to think. Two weeks ago I operated upon a gentleman for appendicitis, who lost a son three months before from this disease without an operation. He presented a history of having had repeated attacks of colic for eighteen years. He said he had never had an attack of appendicitis to his own knowledge. On the morning of the operation I saw him in consultation with his family physician with a normal temperature and pulse, but pain in the region of the appendix, this attack differing from previous ones. Appendicitis was diagnosticated, the patient was sent to the hospital, and I operated on him the next day. I evacuated fully a teacupful of pus and found a gangrenous appendix.

In regard to foreign bodies, in one case I found a piece of chewing gum; in another a piece of wax, and in a case from Buffalo, I found a cherry stone which the patient had swallowed a week or ten days before, that had gotten into the appendix. From the moisture and heat it had swollen and torn a hole into the peritoneum.

Dr. J. B. Murphy, Chicago: This battle of appendicitis has been contested all along the line. The first thing we had to defend was the presence of pus in these cases. Almost every general practitioner that you discuss the subject of appendicitis with would say "There was no pus in my case. The patient got well." Finally, after careful examination at the post-mortem table, it is practically agreed that in every case of appendicitis there has been, or there is present, pus. What will be the outcome of that pus? You know the way in which Nature performs so-called cures, or takes care of this disease. Every general practitioner will tell you how many cases he has had with recovery. At a meeting in one of our western cities this summer, a practitioner who lived in a hamlet of two or three hundred inhabitants had sixty-two recoveries from appendicitis. They were not of the variety that come under my observation.

A word with regard to the pathology. We agree that some cases get well without operative interference. What cases will you, and what cases will you not, operate on? These are two things we want to settle. What shall we do with a man in his first attack of appendicitis? Shall we wait? That is the question. In his first attack, what happens? He has his first symptom either from a perforation, from an invasion with infection of the mucous membrane, which is the most common, or from an obliteration. The outcome of these three things will be all the pathological conditions that you can possibly imagine almost in the peritoneal cavity. In the early stage, what have you to contend with? By the early stage I mean at the time when the patient has his first symptom. You have a disease at this time that is limited to the cavity of the appendix, while a few days later—many times a few hours—it has no limitations except the peritoneal cavity. What would you do with pus of that dangerous variety anywhere else? Of course, you would cut down, evacuate it, and try to save the life of your patient. When a patient has unmistakable symptoms of appendicitis, not tomorrow, not today, but *now* is the accepted time to operate. The symptoms of the disease are more definite and less liable to mislead the surgeon in the early stage than the symptoms of any one affection I know of, excepting pneumonia. We must not delay operative interference until tomorrow, but resort to it at once. Whenever you have a patient with a sudden attack of pain in the abdomen, with nausea and vomiting, increased local tenderness over the seat of the appendix, with perhaps a rise of temperature, you may conclude that you have a case of appendicitis to deal with. There are very few conditions in the abdomen resembling that. Indeed, the exceptions are so few that we can lay down a rule except in diseases of women where there has been a previous history of trouble. Even if you have symptoms on that side of the abdomen in a woman, I think the indications are just as great to operate as in the appendix. As soon as you have the symptoms I have mentioned, you should at once proceed to prepare your patient for an operation. It has been stated that I would operate on such and such a case, but that I *would not* operate on this or that case. Let me say here, that there is no man living who can make a differential diagnosis of the pathological conditions that exist inside the abdomen in appendicitis. Take a case tonight with a temperature of 99½°, the abdomen soft, with a pulse of 82, what is in that abdomen? The patient had an attack of pain, nausea and vomiting yesterday morning, which was Saturday we will say, went home and rested a few hours, got up Sunday morning at 8 o'clock, and went to a drug store to get something to relieve the uncomfortable feeling he experienced. He had a temperature after which the doctor came, who saw him for the first time Sunday afternoon at 3 o'clock. A diagnosis of appendicitis was made. That is all there was; no other symptom to guide him. What was the condition of the abdomen? It was full of pus; fecal concretions had escaped from the appendix on Sunday morning and the contents of the appendix with pus into the abdomen. Until you can make a diagnosis and say of this individual case that there is such and such a condition in the abdomen, you have no right to say I will not operate on that case. You have no right to say I will wait until tomorrow; neither have you any right to hold a patient's life in jeopardy when he has unmistakable symptoms of the disease under discussion, but from a considerable experience and the large number of cases I have had, I should unhesitatingly and immediately resort to surgical interference.

Dr. J. Henry Carstens, Detroit: Dr. Murphy has said nearly everything I wanted to say. I perfectly agree with what he has said, but I must differ from Drs. Morris and Price with reference to one point. There are cases where there is no earthly need of going down and breaking up adhesions, removing the appendix and so on. I am fully in accord with Dr. Cordier's remarks that there is a vast difference between pus from the gonococcus and that which we get from the bacillus coli communis. That is not the only thing; we have sometimes a streptococcus in there.

I desire to emphasize the point brought out by Dr. Murphy, and for which I have been criticised in my own city—namely, that in the present state of our knowledge we cannot always tell whether we have a severe or mild case of appendicitis to deal with. As has been said, ninety-eight times out of every hundred, every case of appendicitis ought to be operated on—not tomorrow, not the next day, but this very minute. If this is done, in the future we will not have statistics giving us thirty-one deaths out of fifty cases, but we will have such statistics as Dr. Morris gives—namely, ninety-eight recoveries out of one hundred operations.

Dr. E. W. Cushing, Boston: I agree fully with what Drs. Murphy and Carstens have said. I simply wish to call attention to one point in Dr. Peck's paper about a secondary operation for freeing the bowel. I would be glad to have this point referred to in the further discussion of the papers, because I think one of the most important things in the whole subject of laparatomy is whether we can do anything for the patient after the operation in relieving bowel obstruction—whether we shall open the abdomen again.

Dr. W. E. B. Davis, Birmingham, Ala.: Reference has been made to purulent peritonitis as being dangerous in connection with appendicitis. We are told that the question in regard to the appendix and tubal disease is settled, and now we are to decide what to do with this affection. I think if there is anything to do, it would be to prevent it. When we get general suppurative peritonitis, it is my opinion that we can do nothing for our patient. We have a local condition of sepsis that will kill the patient, even though we may relieve the general condition; therefore, I think the treatment of purulent peritonitis is preventive. It is the only treatment that we can succeed with. Cases have been reported of suppurative peritonitis; they are not cases of purulent peritonitis, but cases of large abscesses which have ruptured into the cavity, which have been cleaned out before there was peritonitis which could have produced these abscesses in the general cavity.

In regard to breaking up adhesions, I will speak briefly of the treatment of appendicitis. It must be borne in mind that the most dangerous kind of appendicitis, that produces peritonitis in twelve hours, also produces the same condition that we get in stab wounds of the intestines. You all recognize the fact that you cannot save these cases unless you operate within the first day. After twenty-four hours there is but little hope for the life of the patient from operative interference, so much so that it is unsafe (if the patient is seen after twenty-four hours) to operate; in other words, there is no use in operating. The surgeon should take that position in order to sustain his reputation. We must not expect to save many cases of the dangerous form of appendicitis that require our help. Many of the cases that need our help are cases of fulminating appendicitis, in which death comes from obstruction of the bowels. I am sure it has been the experience of all of you that in the majority of cases in which you are called upon to operate on the bowels, they do not have invagination, but obstruction due to dynamic causes. Paralysis and inflammation of the bowel, not due to obstruction of the bowel from mechanical sources, is not an uncommon thing to meet with. The patients that most need an operation do not call us in time to operate. The cases of appendicitis that we see on the second or third day we can usually save, because the pus is circumscribed. We have the existence of a condition with which the surgeon can deal with the promise of a cure. We ought to operate early, but the fact remains that we can save some of these cases late. The cases that need our services most are the ones we do not see to operate on with any degree of certainty of saving them.

In regard to removing the appendix and breaking up adhesions, I think Drs. Morris and Price have advocated a dangerous practice. They have recommended a system of treatment which will, if adopted, cause many deaths. While it may be successful in careful and skilful hands, in the majority of instances it would be a dangerous procedure. I can conceive of nothing so dangerous as allowing the smallest quantity of this offensive septic pus to escape into the abdomen. In the last year, by gentle manipulation of the abscesses, I have had two secondary abscesses produced by the escape of pus, and the patients came very near dying. If you do not find the appendix by very careful and gentle manipulation, you had better let it alone.

Reference has been made to foreign bodies being very frequent causes of appendicitis at certain times of the year. I think we will find the cause is due more to a catarrhal condition, brought about by eating too much fruit, than from the seeds themselves.

Dr. A. Vander Veer, Albany, N. Y.: I have been very much interested in the remarks that have been made with regard to appendicitis, as I have done quite a number of operations for this disease. Dr. Peck has placed on record cases that, if we study them carefully, will be of value to us and to him. I have listened to this discussion with a great deal of interest, because I know it will be published in our medical journals and will be taken either one way or the other—as a scientific discussion by men who understand what they are talking about, or else it is to be looked upon as being extreme. We greatly respect the opinion of such men as Drs. Morris and Price, men who are not only scientific in their attainments, but whose results are above that which many of us can reach. As has been remarked by Dr. Davis, I do not believe it is possible to cure septic peritonitis in which we have the bacillus coli communis. We have the perforative and fulminant forms of appendicitis, that go on and are precisely like gunshot or stab wounds. If we could reach these cases early enough, lives might be saved, but how many surgeons reach the patient in time to perform a life-saving operation? These are the cases that we ought to dwell upon, and talk more about, and instruct the general practitioner, and have him thoroughly educated as to what the symptoms mean.

As to foreign bodies, I do not think this is a matter of so much importance, but fecal concretions are found in a great many cases. I have a number of specimens that I have preserved, but I believe with Dr. Price that at certain periods of the year, during the blackberry and grape season, more cases of the disease are liable to occur. The cecum is filled with seeds. It does not hold impacted feces particularly, but it holds foreign substances in such quantity as to produce irritation of the mucous surface of the appendix, and by extension of the irritative or inflammatory process we meet with a greater number of cases at the time of the year when fruit and certain forms of vegetables are eaten with greater freedom. A word about the cases in which we do not find foreign substances—they are cases of the catarrhal form of the disease. In the process that takes place, the patient is in great danger and we may have a simple form of appendicitis or even perforative appendicitis.

As to the time of operating, I am an advocate of early surgical interference, although I have operated on six cases of relapsing appendicitis without a death.

Dr. Chas. A. L. Reed, Cincinnati: I wish to emphasize one point which has been raised incidentally in the papers and in the discussion. The point is illustrative of some recent cases. I will simply mention one or two. I was called to a town in Kentucky, a few miles below Cincinnati, by an intelligent physician to examine a case that presented a classical history of appendicitis. The doctor had been hesitating about the necessity of an operation, although he recognized there was inflammation about the head of the colon. He concluded it was one of those cases the tendency of which is to recover, and the point upon which he deferred operation is the fact that he was unable to discover McBurney's point. There are a great many members of the profession who believe in McBurney's point; they look for it, and they do not call in a surgeon until they think they can find it. I wish to emphasize the fact that this point must not be looked for. There is no occasion to look for it, and the general practitioner who hesitates and searches for this somewhat mysterious and altogether variable symptom of disease is alluring himself and the patient along to a fatal result.

I recently operated upon a man who came to me with relapsing appendicitis. I operated upon him in the midst of the twenty-first attack. He was a stout, well-built man. I could make out no physical symptoms whatever, but the history was classical, and I operated in the midst of the acute symptoms. I found the head of the colon adherent to all the proximal surfaces. Bringing it up, I found where there had been seemingly spontaneous amputation of the appendix and closure of the orifice. I could find no pus about the head of the colon, yet the symptoms in the midst of which I was operating pointed unmistakably to pus. I enlarged the incision, liberated the adhesions, and three inches above the head of the colon I found a pocket containing not more than an ounce of pus which had already perforated the parietal peritoneum and was burrowing into the abdominal wall, and within two inches of this and two inches farther remote I found the appendix alive and well, being nourished by its adhesion to the colon. These are the anomalous conditions that point to the fact that no man can tell (as Dr. Murphy has said) the pathological conditions within the abdominal walls. When we wait for positive symptoms; when we wait for a positive diagnosis, we simply welcome our patient into an untimely grave.

Dr. James F. W. Ross, Toronto: I take issue with Dr. Davis with regard to large intra-peritoneal abscesses. I have opened the peritoneum of a little girl from which pus and gas spurted out like soda water out of a soda siphon, and after the operation the patient lived for two or three weeks then died from starvation. The original cause of the trouble was an abscess in the mesenteric glands, which perforated into the abdomen. I made a post-mortem examination and removed a pailful of pus from the child's abdomen. To my amazement, the omentum and intestines were firmly glued together with the exception of the peritoneal cavity which was almost obliterated, and there was nothing but a hole in the drainage-tube for anything to escape. Some of these cases of purulent peritonitis will have a tendency to cure themselves.

With reference to cases of fulminating gangrenous appendicitis, they all die. Dr. Bryant, in his remarks in Albany last February, distinctly stated that he had never seen a case of what he called fulminating gangrenous appendicitis but that teiminated fatally. My own cases have all died, and, for that reason, when they come to me in what I call the second stage, I do not operate on them. I do not think any of us can disagree with Dr. Carstens, that when a patient reaches the third stage in which there is pus walled off, the right thing to do is to let the appendix alone. An operation done in the relapsing stage is not dangerous. I have never lost a case on which I have operated in the relapsing condition. As to immediate operation, advocated by Dr. Murphy, I consider it to be sound doctrine, but here in Toronto, at any rate, we do not see these cases early enough. Dr. Murphy says when we see a man with the classical symptoms of appendicitis we should operate at once, but we do not see them here when these symptoms present themselves.

Dr. L. S. McMurtry, Louisville, Ky.: One cannot but recognize in the remarks of the several speakers who have preceded me the marked advance of knowledge in relation to appendicitis. Four or five years ago many eminent surgeons advocated expectant treatment in all cases until general peritonitis was established, and inveighed against operative interference until the last moment. Now the course so forcibly advocated by Dr. Murphy and others in this discussion, is generally conceded to be the only safe method of dealing with this murderous disease.

Of the many important practical points discussed, I beg to direct attention to one in particular. It is to the importance of doing, in every case that will permit, a complete operation. We have learned the disastrous results of leaving behind diseased tissues and multiple pockets of pus in suppurative salpingitis, and the same principles should be applied in operating for appendicitis as in salpingitis. There are, of course, cases of appendicitis in which ideal surgery is impossible, and in which we must be content merely to open, evacuate and drain as an immediate life-saving measure. But in all cases where the patient's strength will permit, a thorough operation should be done. Adhesions should be separated, multiple posterior pockets of pus should be emptied and the appendix removed with free flushing and drainage. I could report in detail numerous cases coming under my own observation, where prolonged illness and secondary operations would have been averted by carrying out this plan thoroughly in the beginning.

Dr. J. D. Griffith, of Kansas City: I would ask the gentlemen who have spoken, particularly Drs. Morris, Price and Murphy, who have had a large experience in this particular line of work, whether they have noticed that a catarrhal condition of the appendix produces a stricture. A year ago, in an article in the *Annals of Surgery*, Dr. Binnie wrote about appendicular colic as calling for operative interference; and Dr. Carstens, in an article published quite recently, speaks of the same thing. I have operated within the last six months on three cases for appendicular colic, not for suppurative appendicitis, and I have been able to demonstrate satisfactorily to myself that in at least two of the cases there was nothing beyond a stricture except an enlargement of the canal. I could only pass a whalebone guide through the strictured portion. There was marked thickening of the circular and longitudinal fibers of the appendix, showing that the circulation had been interferred with to such an extent that there was the commencement in one of the cases of an active ulcerative process. A collection of mucus beyond the stricture and its escape back toward the colon was the cause of the appendicular colic. It seems to me we have an easy way to account for some of these cases, as in the one cited by Dr. Reed where there was no fecal matter, but the orifice of the appendix was closed. We speak of closure of a stricture in the urethral canal, and why cannot the same thing occur in the Fallopian tubes. Why can we not account for the cutting off of the circulation in this way? I think appendicular colic has come to stay.

Dr. M. Hartwig, Buffalo, (by invitation,) took a conservative stand with reference to appendicitis. While the preceding speakers had shown proof of the great danger attending this disease, still if we take the average cases as they come to the general practitioner, appendicitis was not very fatal. German statistics give about ninety per cent. of recoveries in cases not operated on. He admits that we may have the relapsing form of the disease, and he has had five or six cases of this form occur in his practice. Dr. Macdonald has done a good service by pointing out the difference between the operable and non-operable cases, as the speaker fully believes that such a distinction can be made.

Dr. DONALD MACLEAN, Detroit: First of all, I desire to endorse very heartily the doctrine that has been presented by my friend Dr. Carstens—namely, that when you have appendicitis with adhesions fencing off the abdominal cavity and pus outside, you should open it as you would an ordinary abscess and wash it out. But not even the eloquence of Dr. Murphy, the logic of Dr. Price, or the experience of the members of this Association, would induce me to break up the adhesions and enter the peritoneal cavity. I am firmly settled on that point in my own mind.

As to the question of early operation, if I had my patients entirely under my control in the hospital, I certainly would advise and advocate early operative interference. Let us take, for example, a hundred cases in which an early operation is done for appendicitis, and I believe a great many of them would have recovered without an operation and permanently. I have had quite a number of cases under my observation and the opportunity of watching them. One was a coachman who had an acute severe attack of appendicitis, but was unwilling to have an operation performed. He got well without it. I have watched ever since and he is well today. I could cite many other cases, if necessary. In our practice we have to contend with the opposition of the friends or relatives of the patients regarding surgical interference, and this is a serious matter. It takes a man with a good deal of moral courage to go into a family and say to a mother or father, "your lovely daughter of fourteen has got appendicitis and must be operated on at once." It causes the family great sorrow, and sometimes catastrophies of another kind result from it. For instance, in Detroit, a young lady of a prominent family was taken with appendicitis and so diagnosticated by her physician. He called in a surgeon, who advocated immediate operation, which had the effect of horrifying the family to such an extent that my surgical friend and physician were both dismissed from the case. A homeopath was called and sure enough the case got well.

Dr. W. G. Macdonald, Albany: From what I have seen, the operative treatment of appendicitis has been largely confined, among a great many operators, to two classifications—cases of localized abscess with plastic peritonitis, and cases of relapsing appendicitis. Of all cases of relapsing appendicitis upon which I have operated, every one of them has gotten well, and of the other class of cases there is a mortality of 28 per cent. Five of these cases can be attributed to the breaking up of adhesions and establishing a communication between the general peritoneum and this area of the abdomen which has been suffering. I do not believe there is any system by means of which you can make it safe to turn this portion of infected peritoneum into the general peritoneum again, drainage or no drainage, or by any system of drainage.

Dr. Joseph Hoffman, of Philadelphia: Why gonorrheal pus is less irritating than that from the coccus which is supposed to inhabit the colon, is beyond my understanding. It is only an effort on the part of some to explain what we cannot understand, and the explanation does not go far, for the reason that we can constantly open up these colonic abscesses and they get well. There is no reason why, if the coccus of the colon is more poisonous than any other, one patient should get well and another should not, if there has been an abscess opening into the peritoneum. A case was cited in point.

